# Dynamic modeling of PISA achievement scores: comparative analysis of artificial neural networks and differential equation systems approaches

**DOI:** 10.1038/s41598-025-32456-2

**Published:** 2025-12-22

**Authors:** Bahatdin Daşbaşi

**Affiliations:** Faculty of Engineering, Architecture and Design, Department of Engineering Basic Sciences, Kayseri University, Kayseri, Türkiye

**Keywords:** Artificial neural networks, Differential equations, Sensitivity analysis, Educational modeling, PISA, Engineering, Mathematics and computing

## Abstract

In this study, the effects of nine key educational problems in schools on student achievement in mathematics, science, and reading in the Programme for International Student Assessment (PISA) were examined using artificial neural networks and differential equation systems. A linear differential equation model was used to describe instantaneous changes over time for twelve variables. Furthermore, the structure in which the nine education-related variables were defined as inputs and the PISA scores as outputs was analyzed through a neural network model with a tangent hyperbolic activation function. Model parameters were estimated using a least-squares curve fitting procedure, and numerical simulations were implemented to evaluate model accuracy. The performance of the models was evaluated using statistical metrics such as root mean square error, sum of squared errors, and the coefficient of determination. Both approaches yielded high accuracy, which is expected given the low variability and aggregated nature of the national-level indicators; however, the neural network model produced slightly lower error levels and a stronger overall fit. Fractional-order differential equation simulations were also explored; however, they did not provide performance improvements relative to the ODE and ANN approaches. In addition, the effects of factors related to problems in education on each PISA score were determined using a neural network–based sensitivity analysis, and the most influential variables were reported. Finally, short-term scenario-based extrapolations of PISA scores for Türkiye were generated using the differential equation model, purely as mathematical extensions of the fitted trends.

**Supplementary Information:**

The online version contains supplementary material available at 10.1038/s41598-025-32456-2.

## Introduction

In recent years, large-scale international evaluations have become indispensable tools for evaluating and comparing global education systems. The Programme for International Student Assessment (PISA), launched by the Organisation for Economic Co-operation and Development (OECD), is one of the most important of these initiatives. Unlike curriculum-based tests, PISA assesses 15-year-old students’ abilities in mathematics, science and reading, focusing on their capacity to apply knowledge to real-world situations and solve unfamiliar problems^1^. Held every three years, PISA provides participating countries with not only performance data but also detailed contextual information, allowing for international comparisons and insights into the factors that influence educational outcomes^2^.

A growing body of research emphasizes the role of school-level factors, such as classroom environment, institutional resources, administrative quality, and student-teacher interaction, in shaping educational outcomes beyond what can be explained by individual or socioeconomic characteristics^3–5^. Moreover, among the many variables known to influence student performance are the availability and quality of learning materials, the level of educational expenditure, the responsiveness of teachers, the accessibility of schools, the registration bureaucracy, infrastructure conditions, and school climate^6–9^. These institutional inputs can support or hinder students’ learning experiences, especially when combined with challenges related to class size or hygiene^10,11^.

This study examines how national PISA outcomes in mathematics, science, and reading correspond to nine school-related structural indicators reported for Türkiye. Therefore, the problems related to education in schools given as nine different input variables are: quality and quantity of educational materials, educational expenses, teachers’ attitudes towards students, registration procedures, transportation and school accessibility, quality of education, school management’s approach, infrastructure and hygiene conditions, and class size and student density. The three different outcome variables are PISA scores in the following areas: math, science, and reading. This manuscript uses a dual-method approach integrating ANN (Artificial Neural Network) and ODE (Ordinary Differential Equation) systems to examine the complex relationships of the above-mentioned input and output variables.

ANNs are widely recognized for their ability to model nonlinear and high-dimensional patterns without assuming a specific underlying data distribution^12,13^. On the other hand, Ordinary differential equations (ODEs) are commonly used to represent smooth changes in aggregated systems. This mathematical form, used to describe dynamic events and evolution, has been used successfully for centuries in many different fields, from social sciences to natural sciences and biochemistry^14^. In this study, the ODE formulation serves as an exploratory mathematical tool for approximating national-level trends, without implying individual-level temporal dynamics or causal mechanisms. Although less commonly used in educational modeling, ODEs provide a valuable tool for simulating structural interactions and system feedback. Thus, this interdisciplinary research combines machine learning, educational science, and mathematical modeling to provide a comprehensive approach to understanding how structural school conditions influence student learning outcomes internationally.

Although the dataset is aggregated at the national level, ANNs provide a flexible approximation tool for detecting potential nonlinear associations among the indicators. This complements the ODE-based formulation within a unified analytical framework, without implying deterministic relationships. The comparison with ODE modeling provides a complementary perspective, allowing us to evaluate both data-driven and equation-based approaches under the same conditions. This combination enables us to assess how flexible models and theoretically grounded formulations perform when modeling educational performance trends over time.

The aim is to introduce a new methodological framework to the field of educational evaluation by combining the data-driven power of ANNs with the mechanistic insight of ODEs. The primary objectives are to (1) identify the school-based factors that most significantly influence PISA performance, (2) develop and compare ANN- and ODE-based models of student achievement, and (3) provide actionable recommendations for policymakers and educators.

This study makes several novel contributions to the literature. First, unlike previous studies that rely almost exclusively on statistical or machine learning techniques, this research integrates differential equation systems with artificial neural networks to analyze national-level education indicators. Second, while existing PISA studies are typically cross-sectional, our approach provides a continuous-time approximation of aggregated national indicators, without implying actual temporal dynamics or longitudinal evolution. Third, The exploratory use of fractional-order equations is presented only as a mathematical extension, without implying memory-dependent learning dynamics. Finally, the study combines policy-relevant national indicators from TurkStat with PISA performance data, providing a robust and interdisciplinary methodological framework that can be adapted for other countries as well.

The remainder of this paper is organized as follows. Section "Literature review" presents the literature review and summarizes related studies in education with respect to ANN and ODE mathematical modeling. Section "Methodology" describes the dataset, variable selection, and the methodological framework, including both artificial neural network and differential equation approaches. Section "Findings" reports the empirical findings, including model estimation results, performance metrics, and effect analysis. Finally, Sect. "Results and discussion" provides the discussion and interpretation of the results.

## Literature review

Although differential equation–based modeling is uncommon in PISA research, a small number of studies have applied ODE or FODE approaches to broader educational contexts. Most existing PISA studies rely on traditional statistical models or machine-learning techniques, particularly artificial neural networks (ANNs), which have been widely used to analyze large-scale assessment data. A selection of representative ANN- and differential equation–based studies in the field is summarized in Table 1.

**Table 1:** Representative studies employing ANN and differential Equation–Based approaches in educational Research.

Reference	Country/Region	Methods	Main Findings
Koyuncu^15^	Türkiye	ANNs and Linear Regression	Using data from PISA 2003 and 2012, the factors affecting Turkish students’ mathematics achievement were examined. The analysis, conducted with artificial neural networks, found that the factors that most significantly affected achievement were students’ mathematics self-efficacy, interest, motivation, teacher support, and student-teacher relationships.
Bozak and Aybek^16^	Türkiye	Logistic Regression and ANNs	In the study conducted according to PISA 2015 data, it was concluded that although artificial neural networks were found to be statistically more successful than logistic regression in predicting the science achievement of Turkish students, there was no significant difference between the two methods in practice.
Govorova et al.^17^	OECD	Network Analysis	According to PISA 2018 data, self-efficacy, fear of failure and a sense of belonging play a central role in well-being, while teacher support has been shown to be effective in creating a positive school climate and reducing bullying.
Toprak and Gelbal^18^	Türkiye	ANNs, Decision Trees, and Discriminant Analysis	Among artificial neural networks, decision trees and discriminant analysis methods, artificial neural networks showed the highest performance in classifying the mathematics achievement of Turkish students, while sample size and variance homogeneity significantly affected the success of the methods.
Benzer and Benzer^19^	OECD	ANNs	Using PISA science level data from Türkiye and OECD countries, an evaluation was made with a two-layer feedforward artificial neural network with a tan-sigmoid activation function in the input-output layers, and the results showed that the artificial neural network approach provided higher accuracy than regression methods.
Güre et al.^20^	Türkiye	Radial Basis Function Neural Network	Among the factors affecting students’ mathematics achievement in Türkiye, family socioeconomic status, teacher qualifications, school resources and students’ learning attitudes play an important role.
Özkan^21^	A total of 15 OECD and non-OECD countries	Multiple Linear Regression and ANNs	Research using PISA 2018 data showed that students who are interested in environmental issues have significantly higher scientific literacy scores than students who are not interested.
Güre et al.^22^	Türkiye	Multi-layer Perceptron, ANNs and Random Forest Algorithm	The study, which emphasizes the importance of data mining in education, showed that the factors that most affect mathematics achievement are anxiety level, Turkish success level, mother’s education level, motivation, epistemological beliefs, teacher interest and classroom disciplinary environment.
Aksu et al.^23^	Singapore, Japan, Norway, USA, Türkiye, Dominican Republic	ANNs and Decision Trees	Using 2015 PISA data, mathematics achievement in Singapore, Japan, Norway, the United States, Türkiye, and the Dominican Republic was analyzed using M5P decision trees and artificial neural networks. Socioeconomic status was the most influential factor in most countries, while in Japan, years of learning mathematics and father’s education level were the most prominent. Prediction accuracy varied across countries, with Singapore (86%) showing the highest and Türkiye (26%) showing the lowest. The results suggest that education policies should be tailored to the individual country.
Uzun et al.^24^	-	FODEs	In this study, students’ academic success was divided into three groups according to GPA, taking into account individual, family and school factors, and mathematically modeled with fractional differential equations; the results obtained showed that motivation, family support and positive school environment play a critical role in increasing success and that the model helps schools develop preventive strategies.
Ramponi and Tessitore^25^	-	ODEs	This study analyzes the impact of education programs in prisons using a mathematical model, assuming that crime spreads through social interaction. The transitions between individuals in three groups are modeled using differential equations, and the equilibrium states of the system and the potential for crime to spread are examined. The results demonstrate that, if implemented appropriately, education programs can play a significant role in reducing crime rates.
Kandemir^26^	-	ODEs	Disciplinary, teacher, negative, and student factors affecting educational success were examined using mathematical models. The time-dependent effects of these factors on achievement were analyzed using differential equations. Consequently, the effects of these factors on achievement and their dynamic changes were revealed, and the areas that should be focused on to increase educational success were determined.

While hybrid ANN–ODE frameworks are more common in physical, biological sciences or engineering problems^27–31^, their methodological structure provides a useful conceptual foundation for exploratory modeling. This study adapts this general framework to the educational context with appropriate caution regarding the cross-sectional nature of PISA data.

This study presents a hybrid methodology that integrates ANNs with ODE systems to examine associations between school-related indicators and PISA performance. ANNs provide a flexible structure for capturing nonlinear patterns, while the ODE component offers a mathematical approximation of national-level trends without implying true temporal dynamics. Using both approaches on the same dataset provides complementary perspectives for understanding aggregated educational indicators.

## Methodology

In this study, the impact of certain educational problems in Türkiye on PISA scores was analyzed using both ANN and continuous-time modeling. The variables used are presented in Table 2.

**Table 2:** State variables.

Variables	Symbol	Definition
**Input Variables** **(Problems Experienced in Education (%))**	$\:{x}_{1}$	Quality/Number of Educational Tools
	$\:{x}_{2}$	Education Expenses
	$\:{x}_{3}$	Teachers’ Approach to Students
	$\:{x}_{4}$	School Registration Procedures
	$\:{x}_{5}$	Transportation to School/Services
	$\:{x}_{6}$	Quality of Education in School
	$\:{x}_{7}$	General Approach of School Administration
	$\:{x}_{8}$	School Heating, Cleaning, etc. Conditions
	$\:{x}_{9}$	Student Density in Classes
**Outcome Variables** **(PISA Scores)**	$\:{y}_{1}$	Mathematics
	$\:{y}_{2}$	Science
	$\:{y}_{3}$	Reading

### Data sources and collection

The output variables used are data from PISA, administered by the OECD. PISA is a triennial international survey that assesses the proficiency of 15-year-old students in mathematics, science, and reading. It provides a standardized and robust measure for comparing educational outcomes across national contexts. The PISA variables used in this study are the average national PISA scores for Türkiye from seven assessment cycles spanning 2003 to 2022. Furthermore, this study includes nine educational input variables hypothesized to influence student achievement, complementing the PISA performance data. Thus, data on educational input variables for Türkiye were obtained from TURKSTAT for the years 2009–2023, and data on PISA variables were obtained from the OECD for the years 2003–2022. The input variables are derived from the TurkStat Life Satisfaction Survey, which is a nationally representative household survey rather than a school- or student-level census. The data reflect the opinions of heads of households with children under 18 attending school, rather than direct student responses. Therefore, information on the number of individual students or schools is not directly available. However, each annual wave covers approximately 10,000–12,000 households nationally, representing the school-aged population in Türkiye through stratified sampling.

For the outcome variables (PISA mathematics, science, and reading scores), we use national mean scores published by the OECD. These represent country-level averages based on 5,000–6,000 students in each cycle in Türkiye (e.g., 5,815 students in 2018 and 6,037 students in 2022). Each predictor variable corresponds to a specific problem category in the TurkStat Life Satisfaction Survey. For example, $\:x_{3}$ represents perceived problems with teachers’ approaches to students, and $\:x_{4}$ reflects difficulties during school registration procedures. These indicators are reported annually as national percentages.

Table 3 summarizes the descriptive statistics of all predictor and outcome variables used in this study. The input variables correspond to categories of school-related problems reported in the TurkStat Life Satisfaction Survey for households with children aged under 18. National PISA mean scores were used as outcome variables. All values are expressed as national-level percentages or mean scores for the corresponding year.

**Table 3:** Descriptive statistics.

Variable	Min	Max	Mean	SD	Variable	Min	Max	Mean	SD
$\:{x}_{1}$	27.6	41.5	32.74	4.52	$\:{x}_{7}$	13.5	18.7	16.30	1.69
$\:{x}_{2}$	34.8	58.0	44.35	6.99	$\:{x}_{8}$	7.1	13.8	10.86	1.80
$\:{x}_{3}$	7.2	15.3	11.25	1.88	$\:{x}_{9}$	25.6	45.0	34.09	6.33
$\:{x}_{4}$	7.6	15.2	9.99	1.93	$\:{y}_{1}$	420	454	444.0	13.91
$\:{x}_{5}$	8.8	24.9	17.09	4.89	$\:{y}_{2}$	425	476	457.2	19.69
$\:{x}_{6}$	25.5	33.8	29.43	2.24	$\:{y}_{3}$	428	475	457.8	17.68

The input variables used in this study were obtained from the TURKSTAT Life Satisfaction Survey. These variables cover the years 2009–2023 and refer exclusively to public schools. The output variables, consisting of PISA mathematics, science, and reading scores, correspond to the years 2009, 2012, 2015, 2018, and 2022. These scores represent national averages published by OECD.

### Data preprocessing and integration

Given the different temporal resolutions between the PISA assessments and the annual measurement of education input variables for the analysis, a data alignment procedure was necessary to facilitate integrated analysis. To this end, linear interpolation was applied to the PISA scores, which were issued every three years between 2003 and 2022 (excluding the pandemic year of 2021), solely to construct a continuous annual grid for the ANN and ODE models. These interpolated values are purely synthetic and were not treated as empirical observations in any statistical evaluation, model fitting, or performance assessment. This ensured the alignment of input variables. The dataset obtained for Türkiye is shown in Table 4. This linear interpolation procedure used to construct the annual time grid is defined explicitly in the manuscript, and the detailed step-by-step formulation is provided in the README sheet of the Supplementary_Data.xlsx file for full reproducibility.

**Table 4:** Dataset^32,33^.

$\:\boldsymbol{t}$	$\:{\boldsymbol{x}}_{1}$	$\:{\boldsymbol{x}}_{2}$	$\:{\boldsymbol{x}}_{3}$	$\:{\boldsymbol{x}}_{4}$	$\:{\boldsymbol{x}}_{5}$	$\:{\boldsymbol{x}}_{6}$	$\:{\boldsymbol{x}}_{7}$	$\:{\boldsymbol{x}}_{8}$	$\:{\boldsymbol{x}}_{9}$	$\:{\boldsymbol{y}}_{1}$	$\:{\boldsymbol{y}}_{2}$	$\:{\boldsymbol{y}}_{3}$
2009	41.3	49.1	10.6	10.3	12.5	31.8	17.5	12.2	41.8	445	454	464
2010	41.5	47.9	12.2	11.8	11.6	31.7	18.7	10.9	40.5	446	457	467.$\:\stackrel{-}{6}$
2011	37.5	41.2	10.9	12.2	8.8	30.7	17.5	9.9	36.4	447	460	471.$\:\stackrel{-}{3}$
2012	35.2	39.2	10.8	8.5	8.8	28.4	16.3	11.8	30.8	448	463	475
2013	32.4	38.8	12.1	10.9	17.6	28.5	16.7	12.4	30.6	438.$\:\stackrel{-}{6}$	450.$\:\stackrel{-}{3}$	459.$\:\stackrel{-}{3}$
2014	32.4	38.9	11.3	9.1	17.1	28.6	14	13.8	28.7	429.$\:\stackrel{-}{3}$	437.$\:\stackrel{-}{6}$	443.$\:\stackrel{-}{6}$
2015	29.9	41.1	10.4	10.1	17.3	25.5	14.2	12.5	29.2	420	425	428
2016	27.6	37.1	12	7.6	17.8	27	13.5	10	27.5	431.$\:\stackrel{-}{3}$	437.$\:\stackrel{-}{6}$	440.$\:\stackrel{-}{6}$
2017	28	34.8	12.4	8.9	19.3	29	16.2	13.3	25.6	442.$\:\stackrel{-}{6}$	450.$\:\stackrel{-}{3}$	453.$\:\stackrel{-}{3}$
2018	28.2	44.2	10.5	9.1	18.1	27.4	15	8.8	32.4	454	468	466
2019	29.3	45	12.5	9.2	18.8	29.3	17.4	9.9	32.5	453.75	465.5	463.5
2020	28.7	41.3	7.2	9.4	17.3	30	14.6	7.1	28.3	453.5	468	461
2021	34	53.5	8.3	15.2	23.7	32.1	18.3	10.5	45	453.25	470.5	458.5
2022	32.4	58	12.3	9.6	24.9	27.7	16.4	10.1	38.8	453	476	456
2023	32.3	55.2	15.3	8	22.7	33.8	18.2	9.7	43.2	452.75	475.5	453.5

Table 4 visually illustrates this interpolation process and shows how discrete PISA data points are transformed into a continuous annual series. This methodological choice provides a technical approximation to annual trends and enables the exploratory use of ANN and ODE models on a common time grid. However, the interpolated values themselves should not be interpreted as actual yearly PISA measurements. The dataset here is 15 × 13. In artificial neural network (ANN) modeling, using a sufficient amount of data is crucial for obtaining accurate results. In particular, it is common practice to require the number of data points to be a certain multiple of the number of features to increase model performance and generalization. The literature generally recommends that the number of data points be at least 10 times the number of features^34^. This approach aims to prevent the model from overfitting and increase its generalization capacity. Otherwise, models working with limited data may only fit the training data well and perform poorly on new, unseen data. This is because less data causes the parameters the network learns to become dataset-specific. This can limit the model’s learning capacity, leading to generalization errors^35^. Table 5 details the impact of interpolation, showing the increase from 15 row data points to 141 linearly interpolated data points across all time points and variables. This expanded dataset facilitates the exploratory application of ANN models, but does not create longitudinal information nor real temporal patterns. It is important to note that these interpolated values are not empirical observations, but a numerical construct used to align the annual school-related indicators with the triennial PISA cycles for exploratory modeling purposes.

**Table 5:** Linearly augmented Dataset.

$\:\boldsymbol{t}$	$\:{\boldsymbol{x}}_{1}$	$\:{\boldsymbol{x}}_{2}$	$\:{\boldsymbol{x}}_{3}$	$\:{\boldsymbol{x}}_{4}$	$\:{\boldsymbol{x}}_{5}$	$\:{\boldsymbol{x}}_{6}$	$\:{\boldsymbol{x}}_{7}$	$\:{\boldsymbol{x}}_{8}$	$\:{\boldsymbol{x}}_{9}$	$\:{\boldsymbol{y}}_{1}$	$\:{\boldsymbol{y}}_{2}$	$\:{\boldsymbol{y}}_{3}$
2009	41.3	49.1	10.6	10.3	12.5	31.8	17.5	12.2	41.8	445	454	464
2009.1	41.32	48.98	10.76	10.45	12.41	31.79	17.62	12.07	41.67	445.1	454.3	464.3$\:\stackrel{-}{6}$
2009.2	41.34	48.86	10.92	10.6	12.32	31.78	17.74	11.94	41.54	445.2	454.6	464.7$\:\stackrel{-}{3}$
…	…	…	…	…	…	…	…	…	…	…	…	…
2016	27.6	37.1	12	7.6	17.8	27	13.5	10	27.5	431.$\:\stackrel{-}{3}$	437.$\:\stackrel{-}{6}$	440.$\:\stackrel{-}{6}$
2016.1	27.64	36.87	12.04	7.73	17.95	27.2	13.$\:\stackrel{-}{7}$	10.$\:\stackrel{-}{3}$	27.31	432.4$\:\stackrel{-}{6}$	438.9$\:\stackrel{-}{3}$	441.9$\:\stackrel{-}{3}$
2016.2	27.68	36.64	12.08	7.86	18.1	27.4	14.04	10.$\:\stackrel{-}{6}$	27.12	433.6	440.2	443.2
…	…	…	…	…	…	…	…	…	…	…	…	…
2022.8	32.32	55.76	14.7	8.32	23.14	32.58	17.84	9.78	42.32	452.8	475.6	454
2022.9	32.31	55.48	15	8.16	22.92	33.19	18.02	9.74	42.76	452.775	475.$\:\stackrel{-}{5}$	453.75
2023	32.3	55.2	15.3	8	22.7	33.8	18.2	9.7	43.2	452.75	475.5	453.5

In the ANN analysis, the remaining variables were separated as described above, and the process was conducted in the dataset shown in Table 5. On the other hand, the other optimization method used, modeling using linear differential equations, was analyzed using the data in Table 4, with time as the independent variable for parameter estimation. Both methods aim to estimate three different PISA scores. Additionally, the performances of these methods are compared both for the 15 × 13 dataset in Table 4 and using the original **non-augmented data**.

### ANN method

This study employed ANNs as the primary analytical tool to examine the complex and potentially nonlinear relationships between educational inputs and student achievement. ANNs are computational models inspired by biological neural networks that can capture complex data patterns that traditional linear models may miss^36^. The architecture of the ANN model, consisting of the number of hidden layers, number of nodes per layer, activation functions, and learning rates, is systematically optimized through grid search and $\:k$-fold cross-validation to maximize prediction performance and prevent overfitting. Traditional regression or multilevel models were not adopted because they assume linear and often static relationships between predictors and outcomes. In contrast, the ANN and ODE frameworks allow for capturing nonlinear patterns in national-level educational indicators, without implying temporal or causal dynamics.

In this study, a split-sample approach was used in the ANN process, where the data was divided into 70% for training, 15% for validation, and 15% for testing. This partitioning provided an unbiased assessment of model generalizability and prevented information leakage. Furthermore, the Tanh function was selected as the activation function. In the ANN architecture, which works with 9 input features, produces 1 hidden layer and 3 outputs, the data from the input layer is passed through 9 neurons in the hidden layer, each processed with weights $\:(IW,\:LW)$ and biases $\:({b}_{1},\:{b}_{2})$. The choice of neurons number follows common heuristic rules in neural network design, where the hidden layer size is often chosen close to the number of inputs to reduce the risk of overfitting and ensure stable convergence^37,38^. Thus, the number of neurons in the hidden layer was set equal to the number of input variables (nine) to balance model complexity and generalization. On the other hand, The number of hidden layers in a neural network architecture directly affects the model’s complexity and generalization capability. The universal approximation theorem shows that a single hidden layer with a sufficient number of neurons can approximate any continuous function^39^. Using multiple layers may increase the risk of overfitting while making the training process and analytical interpretation more difficult. Therefore, in this study, a single hidden layer architecture was adopted.

Then, the data coming out of the hidden layer is processed with weights and biases and transmitted to the output layer, which produces 3 outputs. In this study, the activation function investigated in Tanh form is

$$\:\begin{array}{cc}y={b}_{2}+LW\mathrm{tanh}\left({b}_{1}+IWx\right)&\:\left(1\right)\end{array}$$.

where input matrix is $$\:x={\left(\begin{array}{ccccccccc}{x}_{1}&\:{x}_{2}&\:{x}_{3}&\:{x}_{4}&\:{x}_{5}&\:{x}_{6}&\:{x}_{7}&\:{x}_{8}&\:{x}_{9}\end{array}\right)}^{T}$$ and output matrix is $$\:y={\left(\begin{array}{ccc}{y}_{1}&\:{y}_{2}&\:{y}_{3}\end{array}\right)}^{T}$$.

### ODE method

While differential equations are classically used for time-dependent systems, in this study the ODE framework is applied only as a macro-level mathematical approximation of smooth national trends. They are frequently used in social sciences and education research to model how systems evolve. This type of modeling is invaluable for analyzing dynamic processes such as educational systems and student performance. By accurately capturing the interactions of variables and their changes over time, differential equations enable more robust predictions. In education, using differential equation models to examine the effects of variables such as teaching methods, student achievement, and school conditions not only explains the data but also offers important implications for policymaking and strategy development. In particular, observing how multiple factors in education interact and influence each other over time allows for a deeper understanding of the impact of these factors on educational achievement^40,41^. Modeling using differential equations provides in-depth information about dynamic systems and enables important predictions about their future behavior. Such models in education provide powerful tools that teachers, students, and administrators can use to optimize educational processes and make more effective decisions. Systems of linear differential equations are particularly useful when examining linear relationships and interactions and offer significant advantages in analyzing complex dynamic processes. While the PISA data are cross-sectional, the ODE framework in this study was applied at the macrostructural level to model national temporal trends in education indicators. This approach is conceptually similar to population-level modeling in fields such as economics and epidemiology, where aggregate data are used to represent systemic dynamics over time. In this study, the effects of various educational factors on PISA achievement scores were modeled using a system of linear differential equations as a macro-level mathematical approximation. Because PISA scores are cross-sectional rather than longitudinal, the ODE framework does not represent real temporal learning dynamics. It is important to clarify that the ODE framework in this study is not used to model causal learning dynamics at the student level. Because PISA scores are cross-sectional and not longitudinal, the ODE system is employed only as a macro-level exploratory approximation of smooth national trends, without implying continuous or causal temporal evolution. Accordingly, no causal interpretation is attached to the ODE coefficients. The model is evaluated strictly at the actual PISA cycle years (2009, 2012, 2015, 2018, 2022), and its intermediate trajectories from the ODE solution are interpreted only as mathematical interpolations, not as actual year-to-year educational change. No causal or temporal interpretation is attached to the ODE coefficients. With non-negative initial conditions, the general form of the model is:

$$\:\begin{array}{ll}\begin{array}{ll}\frac{d{x}_{1}}{dt}={\theta\:}_{145}+\sum\:_{i=1}^{9}{\theta\:}_{i}{x}_{i}+\sum\:_{i=10}^{12}{\theta\:}_{i}{y}_{i-9},&\:\frac{d{x}_{2}}{dt}={\theta\:}_{146}+\sum\:_{i=13}^{21}{\theta\:}_{i}{x}_{i-12}+\sum\:_{i=22}^{24}{\theta\:}_{i}{y}_{i-21},\\\:\frac{d{x}_{3}}{dt}={\theta\:}_{147}+\sum\:_{i=25}^{33}{\theta\:}_{i}{x}_{i-24}+\sum\:_{i=34}^{36}{\theta\:}_{i}{y}_{i-33},&\:\frac{d{x}_{4}}{dt}={\theta\:}_{148}+\sum\:_{i=37}^{45}{\theta\:}_{i}{x}_{i-36}+\sum\:_{i=46}^{48}{\theta\:}_{i}{y}_{i-45},\\\:\frac{d{x}_{5}}{dt}={\theta\:}_{149}+\sum\:_{i=49}^{57}{\theta\:}_{i}{x}_{i-48}+\sum\:_{i=58}^{60}{\theta\:}_{i}{y}_{i-57},&\:\frac{d{x}_{6}}{dt}={\theta\:}_{150}+\sum\:_{i=61}^{69}{\theta\:}_{i}{x}_{i-60}+\sum\:_{i=70}^{72}{\theta\:}_{i}{y}_{i-69},\\\:\frac{d{x}_{7}}{dt}={\theta\:}_{151}+\sum\:_{i=73}^{81}{\theta\:}_{i}{x}_{i-72}+\sum\:_{i=82}^{84}{\theta\:}_{i}{y}_{i-81},&\:\frac{d{x}_{8}}{dt}={\theta\:}_{152}+\sum\:_{i=85}^{93}{\theta\:}_{i}{x}_{i-84}+\sum\:_{i=94}^{96}{\theta\:}_{i}{y}_{i-93},\\\:\frac{d{x}_{9}}{dt}={\theta\:}_{153}+\sum\:_{i=97}^{105}{\theta\:}_{i}{x}_{i-96}+\sum\:_{i=106}^{108}{\theta\:}_{i}{y}_{i-105},&\:\frac{d{y}_{1}}{dt}={\theta\:}_{154}+\sum\:_{i=109}^{117}{\theta\:}_{i}{x}_{i-108}+\sum\:_{i=118}^{120}{\theta\:}_{i}{y}_{i-117},\\\:\frac{d{y}_{2}}{dt}={\theta\:}_{155}+\sum\:_{i=121}^{129}{\theta\:}_{i}{x}_{i-120}+\sum\:_{i=130}^{132}{\theta\:}_{i}{y}_{i-129},&\:\frac{d{y}_{3}}{dt}={\theta\:}_{156}+\sum\:_{i=133}^{141}{\theta\:}_{i}{x}_{i-132}+\sum\:_{i=142}^{144}{\theta\:}_{i}{y}_{i-141}.\end{array}&\:\left(2\right)\end{array}$$

The $\:{\theta\:}_{i}$ parameters for $\:i=\mathrm{1,2},\dots\:.,156$ in the model were calculated by taking into account the data set in Table 4. For this purpose, the method mentioned in^42^ was used. Thus, these parameters were estimated with minimum error using the lsqcurvefit function, after solving the problem with the MATLAB R2025a using the ode45 solver (Runge–Kutta 4(5)).

The performance of the models (ANN and ODE) was evaluated using multiple metrics: RMSE, SSE and $\:R^{2}$. These metrics provide a comprehensive understanding of the model’s accuracy and explanatory power. Given that the dataset consists of low-variance aggregated national indicators, these metrics especially $\:R^{2}$ must be interpreted cautiously, as their values can be artificially inflated under such conditions.

## Findings

### ANN findings

Considering the augmented data set in Table 5, the weights and biases of the ANN activation function given in Eq. (1) found with the Matlab2025a program are as follows, respectively:

$$\:IW=\left(\begin{array}{ccccccccc}-1.0831&\:1.0018&\:2.6082&\:-3.1518&\:-4.0476&\:2.0770&\:-2.6712&\:5.8939&\:0.7459\\\:5.5662&\:-2.1105&\:-2.6310&\:4.2037&\:3.3309&\:-0.6303&\:-11.7530&\:24.7949&\:-5.1843\\\:3.1450&\:-0.0843&\:12.6574&\:18.4359&\:15.1904&\:-18.9441&\:-27.7878&\:9.2734&\:-2.4347\\\:-2.1163&\:1.1546&\:1.0971&\:1.8784&\:-0.5103&\:2.0116&\:-2.2116&\:0.8222&\:-0.6219\\\:0.1650&\:-0.1251&\:0.0690&\:0.0401&\:0.0232&\:-0.2669&\:-0.1231&\:0.0496&\:0.0663\\\:11.3260&\:1.9314&\:-3.1551&\:17.2876&\:9.5914&\:-10.6115&\:2.1365&\:5.0473&\:-19.7381\\\:3.9553&\:-6.4527&\:2.4116&\:-16.4747&\:21.7489&\:21.5921&\:-25.1998&\:-11.9571&\:0.4894\\\:-0.0649&\:0.1051&\:-0.0733&\:-0.1221&\:-0.0259&\:0.2773&\:0.2662&\:-0.1160&\:-0.0573\\\:6.8209&\:-10.5532&\:8.9931&\:-5.8246&\:0.2230&\:5.2574&\:-9.3166&\:2.4842&\:13.7007\end{array}\right)$$,

$$\:LW=\left(\begin{array}{ccccccccc}100.5905&\:4.2150&\:-80.2885&\:15.2473&\:21.1522&\:-93.8670&\:32.7460&\:33.9952&\:33.1251\\\:101.0060&\:1.0538&\:-123.0794&\:26.1263&\:42.9076&\:-64.3080&\:61.9336&\:48.3843&\:-10.2834\\\:105.7262&\:9.6187&\:-97.8683&\:22.4631&\:52.6831&\:-92.1695&\:29.3879&\:59.2683&\:-9.1840\end{array}\right)$$,

$$\:{b}_{1}=\left(\begin{array}{c}6.8850\\\:10.8745\\\:-10.6747\\\:-19.2283\\\:4.8777\\\:-3.8387\\\:8.8076\\\:-7.7604\\\:-0.7116\end{array}\right)\:\mathrm{a}\mathrm{n}\mathrm{d}\:{b}_{2}=\left(\begin{array}{c}88.0946\\\:104.2701\\\:118.2561\end{array}\right).$$

### ODE findings

Consider the dataset in Table 4. The values obtained when the fixed parameters in system (2) are calculated with minimum error are given in Table 6.

**Table 6:** Rate constants:.

$\:{\theta\:}_{1}=\:-0.17047$	$\:{\theta\:}_{2}=\:\:0.21941$	$\:{\theta\:}_{3}=\:-0.45365$	$\:{\theta\:}_{4}=\:-0.82738$
$\:{\theta\:}_{5}=\:-0.25433$	$\:{\theta\:}_{6}=\:-0.19670$	$\:{\theta\:}_{7}=\:-0.44183$	$\:{\theta\:}_{8}=\:-0.22696$
…	…	…	…
$\:{\theta\:}_{153}=\:-0.00519$	$\:{\theta\:}_{154}=\:-0.01261$	$\:{\theta\:}_{155}=\:-0.00419$	$\:{\theta\:}_{156}=\:-0.00963$

**Fig. 1:**
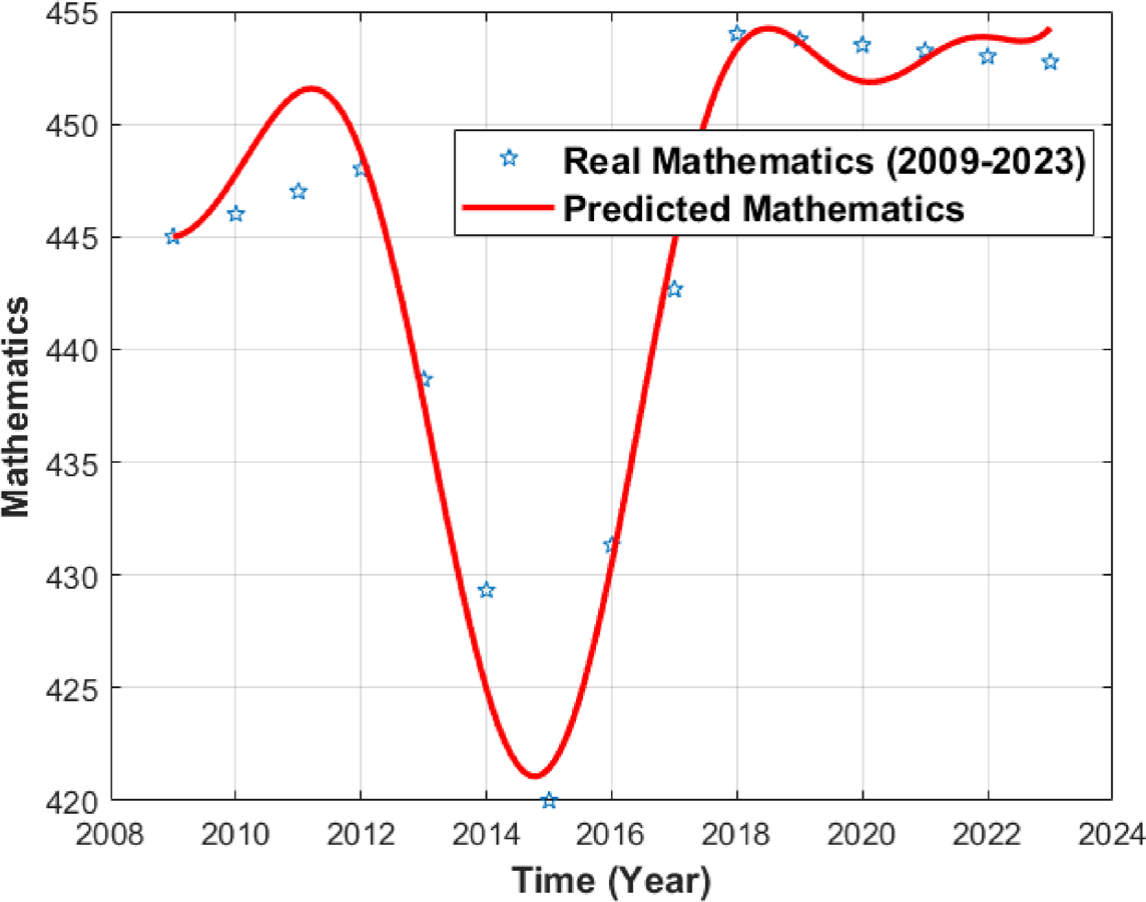
PISA Mathematics Score curve.

**Fig. 2:**
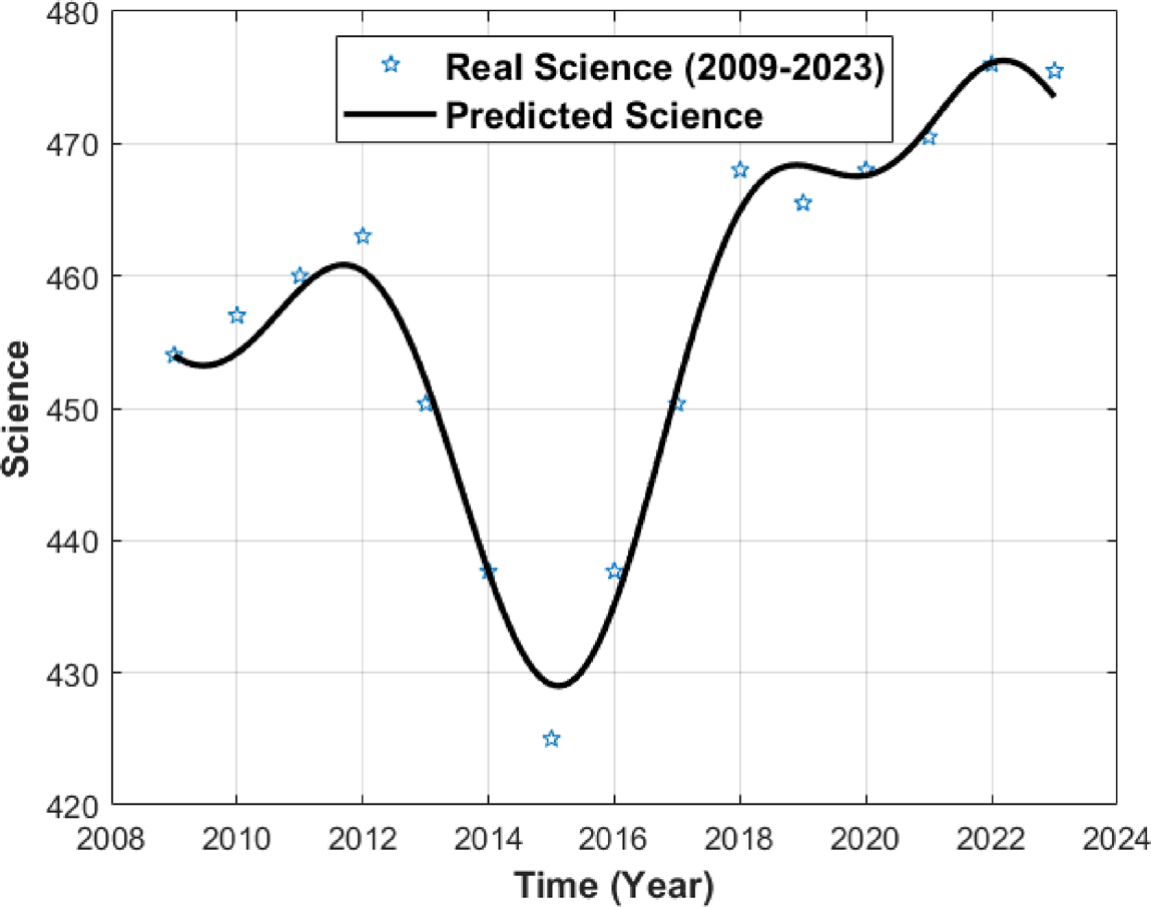
PISA science score curve.

**Fig. 3:**
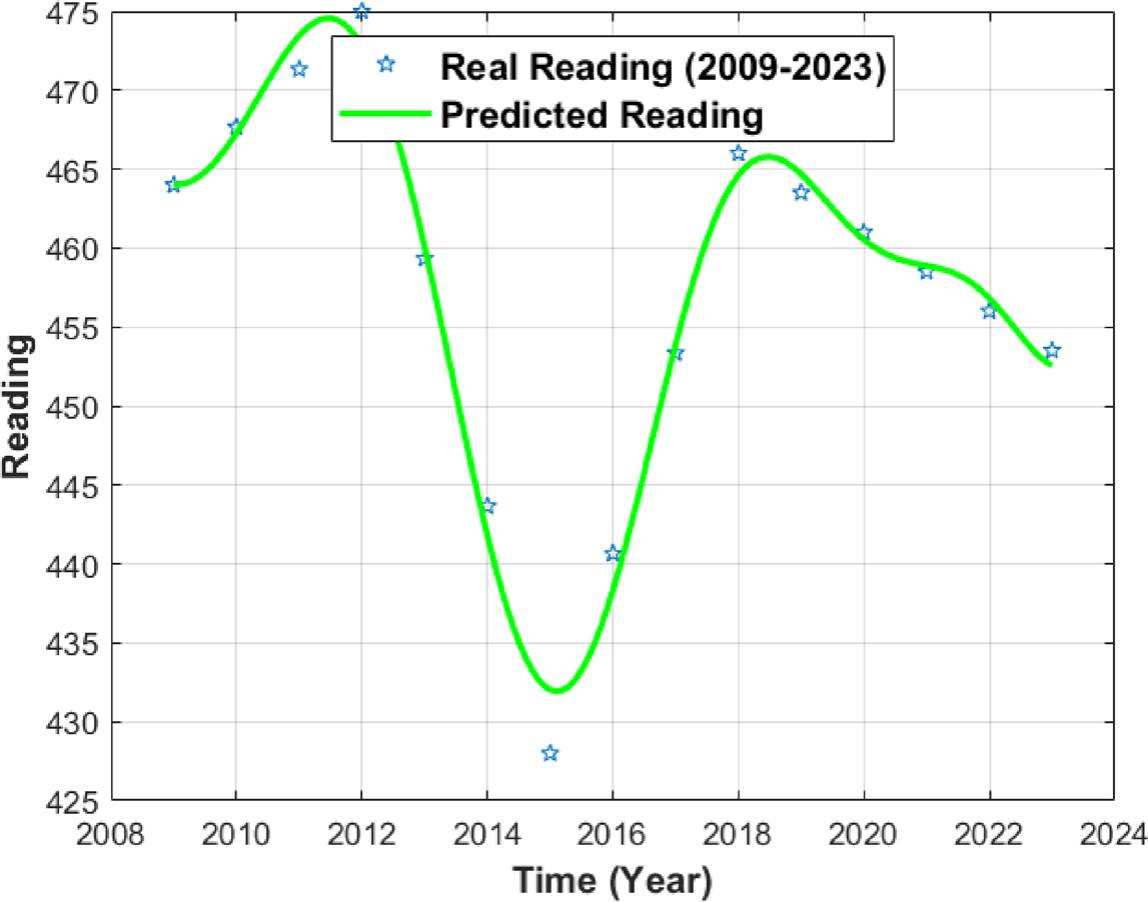
PISA Reading Score curve.

The resulting numerical simulations are given in Figs. 1, 2 and 3.

Figure 1 presents the comparison between the observed PISA mathematics scores at official cycle years and the ODE-generated numerical interpolation across the annualized dataset. The $\:x$-axis represents the constructed annual time grid, and the $\:y$-axis shows the observed PISA mathematics scores at official cycle years alongside the ODE-generated interpolated values. The red line represents the ODE-based numerical interpolation over the annualized dataset, while the markers correspond to the annualized data points, of which only the values at the official PISA cycle years represent actual observations. The overlap between the ODE-generated curve and the observed PISA points reflects numerical consistency on the annualized dataset, without representing real temporal dynamics.

Figure 2 shows the annualized dataset together with the ODE-generated numerical interpolation for PISA science scores. The star markers correspond to the annualized data points, and only those at the official PISA cycle years represent actual observations (2009, 2012, 2015, 2018, 2022). The black line illustrates the ODE-based interpolation across the constructed annual time grid. The visual agreement between the interpolated curve and the observed PISA cycle points reflects numerical consistency rather than real temporal or causal dynamics.

Figure 3 presents the annualized dataset and the ODE-generated numerical interpolation for PISA reading scores. The green markers correspond to the annualized data points, while only those at the official PISA cycle years represent actual observations. The green line shows the ODE-based interpolation over the constructed annual time grid. The visual proximity between the interpolated curve and the observed PISA cycle points reflects numerical consistency, rather than temporal or causal evolution in reading performance. Variations between the cycle years arise from the linear interpolation used to construct the annualized dataset and should not be interpreted as real year-to-year changes.

### Comparison of ODE and ANN methods

Table 7 presents the estimation results of the two optimization methods for the Table 4 values.

**Table 7:** Comparison of the prediction results of two optimization methods with PISA cycle observations (real) and interpolated annual values.

$\:\boldsymbol{t}$	ANN’s Forecast			ODE’s Forecast		
	**Math.**	**Science**	**Reading**	**Math.**	**Science**	**Reading**
**2009**	444.6785	454.1344	464.0879	445.0000	454.0000	464.0000
**2010**	446.1019	456.8956	467.4773	446.8191	455.5964	467.2882
**2011**	446.9016	459.6923	470.9192	449.704	460.3231	473.078
**2012**	447.2179	462.6980	474.6256	448.5849	461.7872	473.4761
**2013**	438.8230	449.7148	458.7118	439.6789	453.2189	461.5718
**2014**	429.3763	437.6563	443.6419	426.7937	437.8244	442.2932
**2015**	419.9476	425.3480	427.959	420.9369	428.3748	430.5688
**2016**	431.4189	437.4216	440.7648	429.0284	434.2097	436.478
**2017**	442.2402	450.6636	453.1128	443.7146	451.3569	453.1724
**2018**	453.8045	467.2851	465.718	453.9327	466.578	465.4592
**2019**	453.5174	465.5729	463.063	454.7342	468.9428	465.3439
**2020**	453.4697	467.9413	460.7507	451.767	466.7414	460.0745
**2021**	453.0189	470.1951	458.5959	451.8146	470.1806	457.8749
**2022**	453.0508	475.4259	456.4937	452.5319	475.3091	456.5886
**2023**	452.7420	475.1953	454.0624	451.3009	474.7102	452.3009

The comparison of the performances of ANN and ODE optimization methods with some metric measurement tools is presented in Table 8.

**Table 8:** Some metric results.

	Prediction Type	Score	RMSE	SSE	$\:{\boldsymbol{R}}^{2}$
Datas in 2009, 2012, 2015, 2018 and 2022	ANN	Math.	0.3895	0.7586	0.9997
		Science	0.4628	1.0710	0.9999
		Reading	0.3075	0.4728	0.9998
	ODE	Math.	0.5373	1.4436	0.9981
		Science	1.7527	15.3595	0.9901
		Reading	1.3827	9.5599	0.9926
Datas in 2009–2023 (Annually)	ANN	Math.	0.2711	1.1026	0.9997
		Science	0.3580	1.9229	0.9998
		Reading	0.3370	1.7040	0.9997
	ODE	Math.	1.4478	31.4435	0.9795
		Science	1.8788	52.9464	0.9830
		Reading	1.6990	43.3003	0.9798

Table 8 presents the performance comparison of ANN and ODE methods under two different scenarios. In the first case, the models were evaluated using the years in which actual PISA results are available (2009, 2012, 2015, 2018, and 2022). In the second case, linearly interpolated PISA values based on the dataset in Table 4 were used, and the models’ performances were compared over the extended period of 2009–2023.

As can be seen in Table 8, while both optimization methods are quite successful in predicting PISA Scores for Türkiye, the ANN method is slightly more successful. It is worth noting that the $\:R^{2}$ values obtained for both models are extremely high, close to 1.0. Because the dataset consists of low-variance, aggregated national indicators, the $\:R^{2}$ values of both models appear very high. These values should be interpreted cautiously, as they primarily reflect the smooth structure of the aggregated data rather than precise predictive accuracy. The ANN model includes standard validation and test partitions, while the ODE model—although formulated as a linear differential system—produces nonlinear solution trajectories due to the numerical integration process. However, neither model structure eliminates the possibility that high R² values may arise from the characteristics of the dataset itself. Therefore, the reported performance metrics should be viewed as descriptive indicators under the given data limitations rather than strong evidence of generalization or model superiority.

### Model generalization assessment

In this section, the consistency and generalization ability of the metrics obtained using the previously defined activation function and corresponding weights were evaluated. In the initial stage, the dataset was divided into 70% training, 15% validation, and 15% test, and the artificial neural network (ANN) model was trained to obtain the baseline performance metrics. This comparison was made with the data in Table 4. This analysis confirmed that the chosen activation function can effectively capture the underlying data patterns.

Subsequently, to provide a more robust evaluation of the model’s generalization capability, the dataset was re-partitioned into 70% training and 30% test, and the same performance metrics were calculated for each output variable separately. By comparing the results across two different data partitioning strategies, the stability and reliability of the model were examined. The results are presented in Table 9.

**Table 9:** Comparison of model performance metrics under two different data partitioning strategies.

	**Output**	Train			Test		
		$\:\boldsymbol{R}^{2}$	**RMSE**	**MAE**	$\:\boldsymbol{R}^{2}$	**RMSE**	**MAE**
**70-15-15%**	**Math.**	0.99968	0.1559	0.1180	0.99795	0.2113	0.1511
	**Sci.**	0.99984	0.1718	0.1371	0.99918	0.2242	0.1859
	**Read.**	0.99978	0.1759	0.1353	0.99887	0.2470	0.2053
	**Avg.**	0.99977	0.1679	0.1301	0.99867	0.2275	0.1808
**70 − 30%**	**Math.**	0.99530	0.3183	0.2366	0.98990	0.1055	0.1022
	**Sci.**	0.99960	0.1263	0.1186	0.99550	0.1654	0.1362
	**Read.**	0.99940	0.1900	0.1633	0.99500	0.2129	0.1961
	**Avg.**	0.99810	0.2115	0.1728	0.99340	0.1613	0.1448

As presented in Table 9, the model exhibits remarkable consistency and robustness across the two data partitioning strategies (70–15–15% and 70–30%).

First, the determination coefficients $\:\left(R^{2}\right)$ remain exceptionally high in both training and test sets (0.9899–0.9998), with minimal fluctuations between partitions. This strongly suggests that the model reliably captures the underlying structure of the data.

Second, the RMSE and MAE values remain consistently low across all output variables, with no meaningful deterioration in the test performance. For the Science and Reading outputs, the test errors are even slightly lower than those in the training set, further supporting the model’s stable generalization capability.

Third, the similarity of results obtained under two independent data partitioning schemes indicates that the observed performance is not sensitive to data splitting, reinforcing the robustness of the ANN model as an intermediate predictive component.

Moreover, the explicit disclosure of weight and bias parameters associated with the activation functions ensures reproducibility and confirms that the high $\:R^{2}$ values are not artifacts of overfitting but are derived from a stable and well-defined functional mapping between inputs and outputs.

Overall, these findings confirm that the model achieves strong generalization, statistical stability, and reproducible predictive performance, and that the high $\:R^{2}$ values should be interpreted as an indicator of model reliability rather than overfitting.

### FODE (Fractional-Order differential Equation) method

**Fig. 4:**
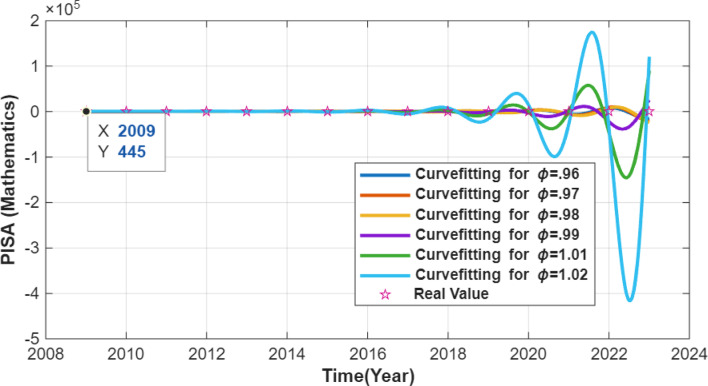
PISA Math. score curves.

**Fig. 5:**
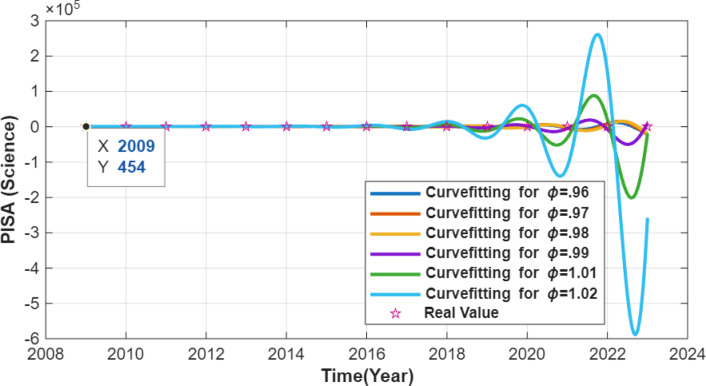
PISA Science score curves.

**Fig. 6:**
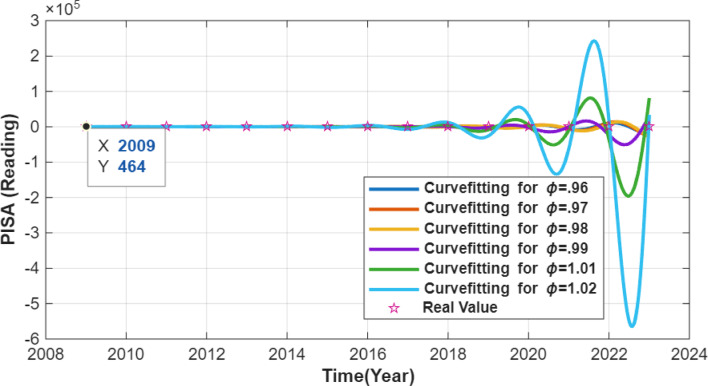
PISA Reading score curves.

In this section, the equation system (2) with the constant parameters obtained in Table 6 is expressed as fractional order differential equations in the Caputo meaning with derivative order $\:0<{\upvarphi\:}\le\:1$. The fractional formulation is used only as a mathematical extension to test whether memory-dependent operators improve numerical approximation, without implying any real temporal memory in PISA data. In this way, it is investigated whether it can give better numerical results than the ODE model. The simulations thus obtained are given in Figs. 4, 5 and 6. In these figures, curves for six different values of the derivative order were obtained and the graphs were compared.

Figure 4 compares mathematics score trajectories obtained from the FODE model under six different fractional derivative orders. The curves show slight variations in their slopes and curvature, highlighting the memory effect embedded in fractional dynamics. Although the FODE trajectories pass near the interpolated PISA trend points at cycle years, this reflects numerical curve-following rather than genuine temporal behavior.

Figure 5 presents the fractional differential equation simulations for science scores. The multiple colored curves correspond to different fractional orders, while the markers represent real observed scores. Although the model approximates real scores reasonably well at PISA years, the diverging behavior between cycles shows that the FODE model does not outperform the ODE model in this context. This suggests that the deterministic national data may not benefit substantially from memory-dependent fractional modeling.

Figure 6 illustrates the FODE-based reading score simulations. The curves represent different fractional derivative orders, while the markers show the observed data. While FODE provides a flexible approximation, its oscillatory behavior between observed data points results in lower predictive stability. The figure supports the conclusion that fractional dynamics add complexity but not necessarily higher predictive accuracy in this dataset.

Consider Figs. 4, 5 and 6. These graphs contain curves for each true value and six different derivative orders. Although these curves produce values quite close to the true values in the integer years between 2009 and 2023, the curve predictions differ significantly between successive years. These differences indicate that fractional dynamics add mathematical flexibility but do not provide meaningful improvement for this low-variance, deterministic national dataset.

### Local sensitivity analysis

The Jacobian-based local sensitivity analysis method proposed by Dimopoulos et al. (1995)^43^ was used to quantify the marginal influence of each input variable on the ANN outputs. To assess the relative influence of each input variable on the neural network outputs, a local sensitivity analysis was performed based on the Jacobian of the network with respect to the inputs. For a given input vector $\:x\in\:{\mathbb{R}}^{p}$, the network output is defined as

$$\:y\left(x\right)={b}_{2}+LW\cdot\:\mathrm{t}\mathrm{a}\mathrm{n}\mathrm{h}\left({b}_{1}+IW\cdot\:x\right)$$.

where $\:IW\in\:{\mathbb{R}}^{h\times\:p}$ is the input-to-hidden weight matrix, $\:LW\in\:{\mathbb{R}}^{m\times\:h}$ is the hidden-to-output weight matrix, $\:{b}_{1}\in\:{\mathbb{R}}^{h}$ and $\:{b}_{2}\in\:{\mathbb{R}}^{m}$ are the bias vectors for the hidden and output layers, respectively. Here, $\:p$ is the number of input variables, $\:h$ is the number of hidden neurons, and $\:m$ is the number of output variables.

The Jacobian matrix of the network is obtained by differentiating the outputs with respect to the inputs: $\:J\left(x\right)=\frac{\partial\:y}{\partial\:x}=LW\cdot\:\mathrm{d}\mathrm{i}\mathrm{a}\mathrm{g}\left(1-{\mathrm{t}\mathrm{a}\mathrm{n}\mathrm{h}}^{2}({b}_{1}+IW\cdot\:x)\right)\cdot\:IW$. The average local sensitivity matrix $\:T$ was then computed by taking the mean of the absolute values of the Jacobian across all $\:N$ data points: $\:{T}_{ij}=\frac{1}{N}\sum\:_{k=1}^{N}{\left|\frac{\partial\:{y}_{i}}{\partial\:{x}_{j}}\right|}_{x={x}^{\left(k\right)}}$where $\:i=1,\dots\:,m$ and $\:j=1,\dots\:,p$. To obtain the normalized sensitivity contribution of each input variable across all outputs, the following index was used: $\:{S}_{j}^{\mathrm{n}\mathrm{o}\mathrm{r}\mathrm{m}}=\frac{\sum\:_{i=1}^{m}{T}_{ij}}{\sum\:_{j=1}^{p}\sum\:_{i=1}^{m}{T}_{ij}}$. The results of the Jacobian-based local sensitivity analysis for the ANN model with $\:p=9$, $\:h=9$, $\:m=3$ and $\:N=141$ are summarized in Table 10, which presents the local sensitivity matrix $\:{T}_{ij}$​ representing the average absolute Jacobian values for each output $\:{y}_{i}$​ with respect to each input $\:{x}_{j}$​.

**Table 10:** Local sensitivity matrix $\:{T}_{ij}$ representing the average absolute Jacobian values for each output $\:{y}_{i}$ with respect to each input $\:{x}_{j}$.

Output	$\:{x}_{1}$	$\:{x}_{2}$	$\:{x}_{3}$	$\:{x}_{4}$	$\:{x}_{5}$	$\:{x}_{6}$	$\:{x}_{7}$	$\:{x}_{8}$	$\:{x}_{9}$
$\:{y}_{1}$	2.8355	1.6872	1.5805	2.7678	0.7213	3.1365	3.5058	1.3442	0.9115
$\:{y}_{2}$	4.9506	2.7790	2.6354	4.6381	1.2105	5.0962	5.7690	2.2005	1.5013
$\:{y}_{3}$	4.3118	2.4469	2.3187	4.1247	1.0599	4.5745	5.2335	2.0139	1.3234

**Fig. 7:**
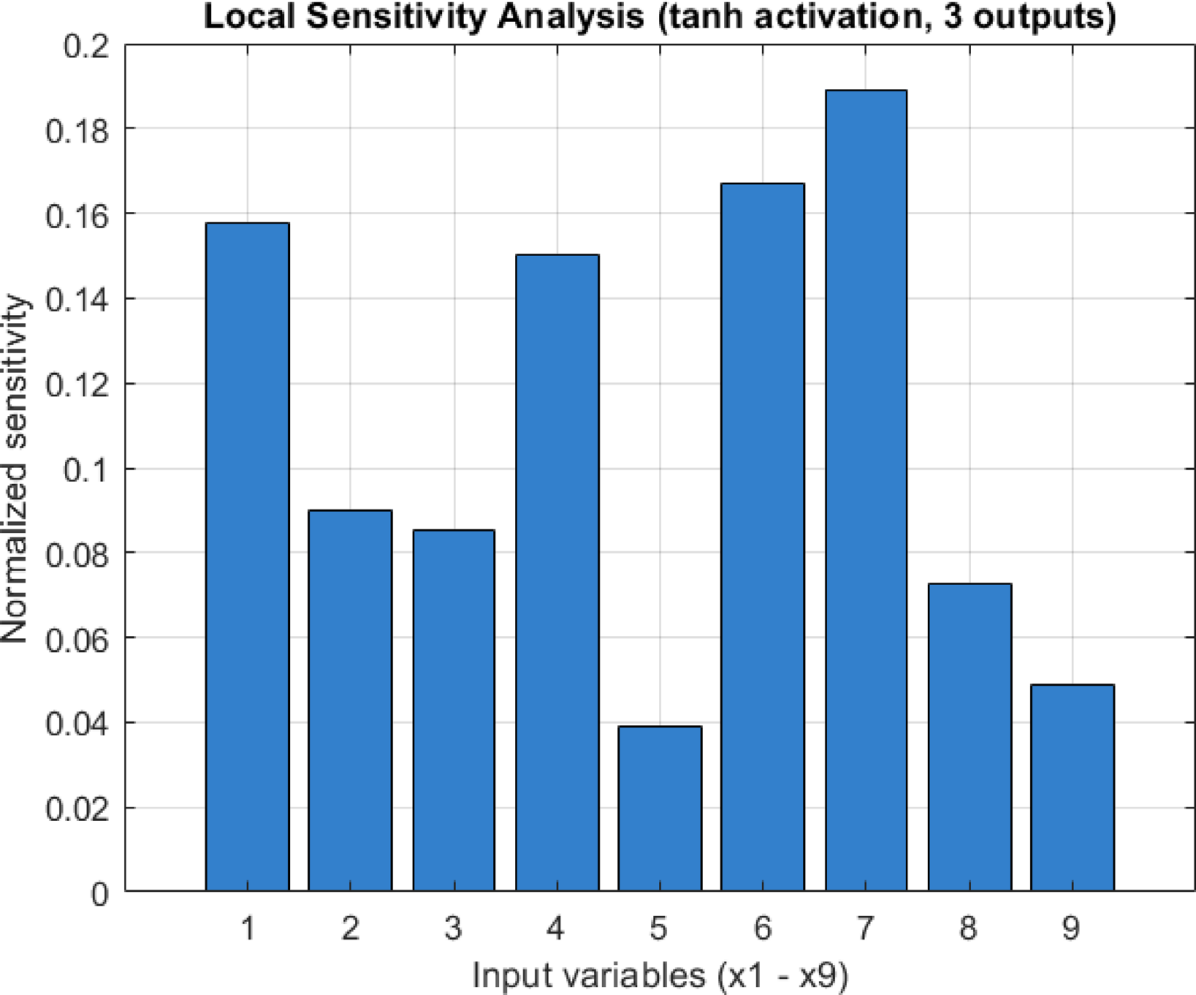
Normalized sensitivity contributions based on jacobian-based local sensitivity analysis.

To provide a clearer comparison of the relative importance of the input variables, the normalized sensitivity contributions derived from the matrix $\:{T}_{ij}$​ are illustrated in Fig. 7.

Considering Fig. 7, normalized sensitivity contributions of the nine input variables ($\:{x}_{1}$–$\:{x}_{9}$) to the three outputs ($\:{y}_{1}$, $\:{y}_{2}$, $\:{y}_{3}$) obtained through Jacobian-based local sensitivity analysis using the hyperbolic tangent activation function. Higher bar values indicate stronger influence of the corresponding input variable on the overall network output. The results show that input variables $\:{x}_{7}$ ($\:18.9\mathrm{\%}$), $\:{x}_{6}$ ($\:16.7\mathrm{\%}$), $\:{x}_{1}$ ($\:15.8\mathrm{\%}$), and $\:{x}_{4}$ ($\:15.0\mathrm{\%}$) contributed most strongly to the network outputs. Variables $\:{x}_{2}$, $\:{x}_{3}$, and $\:{x}_{8}$ exhibited moderate contributions, while $\:{x}_{5}$ and $\:{x}_{9}$ were the least influential. This indicates that the model is particularly sensitive to variations in a subset of input features, which may be prioritized in further feature selection, interpretability analyses, or model reduction steps.

### ODE forecast for 2025–2027

This study examined the educational factors affecting PISA mathematics, science, and reading achievement in Türkiye using both ODE and ANN models. In a performance comparison of the models, the ANN method was found to be superior to the ODE in terms of predictive accuracy. This result demonstrates that artificial neural networks are a suitable tool for modeling the dynamic and complex nature of educational performance. However, differential equations still prevail when it comes to predicting the change of a variable over time. The ODE model’s predictive performance was still quite successful. Therefore, a short-term mathematical extrapolation (3-year) was generated from the ODE model to illustrate the model’s behavior beyond the observed period, without implying actual future PISA predictions. The extrapolated ODE trajectories suggest approximate values for the years 2025–2027, not as forecasts of actual future PISA outcomes but as mathematical extensions of the fitted trend. According to this extrapolation, the continuation of the ODE curve yields values of approximately 494 for Mathematics, 497.4 for Science, and 500 for Reading in 2025. In 2026, the extended trajectories increase to about 511 in Mathematics, 545.5 in Science, and 539 in Reading. In 2027, the extrapolated curve shows a moderate decline, with Mathematics decreasing to 466.5, Science to 540.6, and Reading to 502. These values illustrate only the mathematical behavior of the fitted ODE system beyond the observed period.

These extrapolated values represent only the mathematical continuation of the fitted ODE curve and are not intended as forecasts of actual future PISA results. They do not reflect real-world educational processes or policy effects but illustrate how the estimated ODE system behaves numerically beyond the observed period. The extended trajectories therefore should not be interpreted as predictions of future outcomes; rather, they serve solely as a mathematical demonstration of the model’s functional form outside the data range. Any apparent upward or downward trends in the extrapolated values are mathematical artifacts of the fitted system and do not imply real changes in student performance or the impact of educational policies.

## Results and discussion

The performance comparison results presented in this study evaluate the performance of ANN and ODE-based modeling methods in predicting PISA scores using various metrics. Overall, the ANN method produced lower deviations in terms of error measures and established stronger relationships between predicted and actual values, demonstrating the model’s superiority in terms of stability and accuracy. In contrast, the ODE approach struggled to maintain its prediction accuracy, particularly with increasing data, and error levels a little more increased. In this context, it can be argued that the ANN method is a more reliable and flexible tool for modeling the fitted data structure and capturing nonlinear relationships in the aggregated indicators. Although the ANN model achieved superior performance across multiple error metrics, this superiority is context-specific and reflects its flexibility in capturing nonlinear patterns in aggregated educational data. The ODE model, while less accurate, provides complementary interpretability advantages. Predictor impacts derived from ANN were validated through repeated runs and sensitivity analysis, confirming their stability. The results presented in Table 9 demonstrate that the proposed ANN model exhibits strong generalization and statistical stability across different data partitioning strategies. The determination coefficients ($\:R^{2}$) remain exceptionally high for both the training and test sets, indicating that the model captures the underlying structure of the data with minimal variability. The consistently low RMSE and MAE values across all outputs confirm the model’s ability to maintain predictive accuracy, even when the proportion of test data increases. Notably, the Science and Reading outputs display slightly lower test errors, suggesting that the model does not rely on overfitting but rather on a stable functional mapping between inputs and outputs. These findings collectively highlight the robustness of the model and provide strong evidence that the high $\:{R}^{2}$ values reflect reliable generalization rather than model instability or data partition sensitivity.

Moreover, the effect of input variables on output variables was also analyzed with the ANN method. The models used in the study provide a robust, data-based framework for shaping education policies in Türkiye.

According to the results of the Jacobian-based local sensitivity analysis (Table 10), the input variables with the strongest influence on the network outputs are $\:{x}_{7}$​ (General approach of school administration), $\:{x}_{6}$​ (Quality of education in school), $\:{x}_{1}$​ (Quality/number of educational tools), and $\:{x}_{4}$​ (School registration procedures). These variables exhibited notably higher sensitivity coefficients for Mathematics ($\:{y}_{1}$​), Science ($\:{y}_{2}$), and Reading ($\:{y}_{3}$​) compared to the others. In particular, $\:{x}_{7}$​ and $\:{x}_{6}$​ showed consistently strong effects across all three output variables.

In contrast, $\:{x}_{2}$​ (Education expenses), $\:{x}_{3}$​ (Teachers’ approach to students), and $\:{x}_{8}$ (School heating, cleaning, etc. conditions) displayed moderate effects, while $\:{x}_{5}$​ (Transportation to school/services) and $\:{x}_{9}$​ (Student density in classes) were found to have the least influence on the outputs.

These results indicate that the main factors affecting PISA performance are strongly related to administrative approach, educational quality, and school infrastructure. The high contribution of $\:{x}_{7}$​ underscores the critical role of school climate and organizational factors in shaping students’ academic achievement. Similarly, the high sensitivity of $\:{x}_{6}$ and $\:{x}_{1}$​ suggests that the quality of education and availability of learning tools are directly reflected in student performance.

The finding suggests that improvements in school administration, instructional quality, and educational infrastructure may lead to meaningful enhancements in PISA performance and can guide policy makers and education planners in prioritizing interventions.

Overall, the findings obtained with the ANN model demonstrate that the Turkish education system is shaped not only by structural deficiencies but also by administrative, social, and pedagogical dynamics, and that these dynamics are strong determinants of student achievement. In this context, it is clear that the goal of improving quality in education should not only involve physical improvements but also encompass multifaceted interventions such as enhancing teacher competencies, strengthening school administration, simplifying registration systems, and reducing the economic burden on students. Such holistic and data-driven approaches are crucial for Türkiye’s sustainable improvement in its educational performance on an international scale.

The proposed hybrid modeling framework offers several advantages. First, by integrating artificial neural networks with differential equation systems, it allows for both data-driven flexibility and mathematically interpretable dynamics. Second, the use of national-level educational indicators with low noise levels leads to highly stable model performance. Third, the method provides short-term projections with interpretable parameter structures, which is especially valuable for education policy planning. Finally, the sensitivity analysis embedded in the ANN structure enables the identification of the most influential predictors, providing practical insights for decision-makers beyond pure prediction accuracy.

## Future research

Future research can extend this hybrid ANN–ODE framework in several ways. First, expanding the analysis to include multi-country datasets would allow for comparative modeling across different educational contexts. Second, incorporating additional socio-economic and institutional predictors may help capture more complex interactions affecting learning outcomes. Third, integrating uncertainty quantification and sensitivity analyses will improve the robustness of the results and support more reliable policy recommendations. Finally, applying the framework to real-time or higher-frequency educational indicators may enable dynamic monitoring and early intervention strategies.

## Limitations

This study has certain limitations that should be acknowledged. First, the analysis is based on national-level aggregated PISA data rather than individual student-level data, which limits the granularity of the results. Second, the number of available data points is restricted to five PISA cycles, and interpolation was applied to generate intermediate observations. We fully acknowledge the methodological limitations of this procedure; therefore, model performance was evaluated strictly at actual PISA cycle years to ensure validity and transparency. Third, the model was developed and validated for Türkiye only; therefore, its generalizability to other countries should be interpreted with caution. Despite these limitations, the proposed hybrid ANN–ODE framework demonstrates the feasibility and potential of integrating data-driven and mechanistic modeling approaches in educational performance research. Furthermore, because PISA cycles are cross-sectional, the ODE structure is not assumed to represent real temporal continuity, and all ODE-based results are interpreted with caution as exploratory mathematical approximations. In particular, the linearly interpolated PISA values are synthetic and were used solely to construct an annual time grid for exploratory modeling. Therefore, all results based on the augmented series are interpreted with caution and are not claimed to represent true longitudinal dynamics. Accordingly, the very high $\:R^{2}$ values reported in this study primarily reflect the smoothed structure of the aggregated dataset and should not be viewed as evidence of true predictive strength.

## Supplementary Information

Below is the link to the electronic supplementary material.

Supplementary Material 1

Supplementary Material 2

Supplementary Material 3

## Data Availability

To ensure full reproducibility, all datasets, preprocessing steps, model parameters, and analytical codes used in this study are provided in the supplementary materials. The supplementary Excel file (Supplementary_Data.xlsx) includes: the original PISA scores and TurkStat educational indicators, the annually interpolated dataset used for ANN and ODE modeling, the 141-point interpolated grid used for parameter estimation, and the complete list of ANN weights/biases and ODE parameters (θ₁–θ₁₅₆) used in all simulations. A dedicated README sheet documents all variable definitions, interpolation formulas, preprocessing procedures, and model configuration details. In addition, the full MATLAB scripts used for ANN training, ODE simulation, parameter estimation, and performance evaluation are included for complete transparency. These materials allow any researcher to replicate the entire analysis pipeline and verify all reported results.

## Declarations

### **Use of AI tools**

The author declares that they have not used Artificial Intelligence (AI) tools in the creation of this article.
